# A medical peer-delivered intervention comprising brief motivational interviewing via instant-messaging interaction to reduce drug misuse among youth in Hong Kong: A protocol for a randomised controlled trial

**DOI:** 10.1186/s13722-021-00241-x

**Published:** 2021-05-22

**Authors:** William Ho Cheung Li, Wei Xia, Laurie Long Kwan Ho, Ankie Tan Cheung, Queenie Kuai I. Leong, Tingna Liang

**Affiliations:** 1 School of Nursing, The University of Hong Kong, HKSAR, 4/F, William M. W. Mong Block, 21 Sassoon Road, Pokfulam, Hong Kong, China; 2 School of Nursing, Sun Yat-Sen University, Guangdong, China

## Abstract

**Aims:**

Youth are frequently exposed to drugs, and most youth who misuse drugs are reluctant to seek help from services due to the worry of others being judgmental, lacking expertise, exposing their personal information, or informing their parents. Considering these concerns, we propose to evaluate the effectiveness of a medical peer-delivered intervention comprising brief motivational interviewing via instant-messaging interaction in reducing drug misuse among youth in Hong Kong.

**Methods:**

A two-group single-blind, randomised controlled trial will be conducted. Multiple approaches, including online and face-to-face methods, will be used to recruit the participants. The participants, aged 25 years or younger and reporting any drugs that they have taken within the past 30 days, will be recruited and randomised to receive either brief motivational interviewing via interactive instant-messaging (the intervention) or general health text-messages (comparator). The primary outcome will be the change in the participants’ reductions in self-reported drug consumption at 12 months compared to that at baseline. The secondary outcomes will be the changes in the drug-abusing participants’ reductions in self-reported drug consumption at 6 months, the changes in the drug-quitting participants’ 6- and 12-month contemplation stages and relapse risk compared to that at baseline, 30 days’ self-reported drug abstinence at 6 and 12 months, and the treatment needs and motivation at 6 and 12 months compared to that at baseline. The effectiveness of the proposed intervention will be examined with adjusted regression models, with adjustment for baseline characteristics and the use of an intention-to‐treat approach.

**Discussion:**

This proposed study will be the first randomised controlled trial to assess the effectiveness of a medical peer-delivered interactive intervention to reduce drug misuse among youth in Hong Kong. The proposed intervention has the potential to increase the help-seeking behaviour and intention to quit among youth who misuse drugs. As a result, more youth misusing drugs may be helped to abstain from drugs. This proposed study will inform decisions on whether it is worthwhile to invest resources in large-scale implementation of such an intervention.

## Introduction

Drug misuse is a worldwide problem, especially among youth. In Hong Kong, there is easy access to a wide range of illicit drugs from multiple sources, which has led to an increase in drug misuse among youth. According to the Central Registry of Drug Abuse Report in Hong Kong, 87.3 % of people with a drug use disorder reported that they first used drugs at 25 years of age or younger [1]. Statistics from the Central Registry of Drug Abuse show that the number of students who claimed to have used drugs increased by 23 % between 2014/15 and 2017/18 and that 81 % of those had begun using drugs before 15 years of age [1, 2]. In addition, official figures revealed that 58 % of students who misuse drugs only at their own home or a friend’s home, which was a substantial increase from the 43 % recorded in 2008 [2]. These statistics show the alarming amount and nature of drug misuse and hidden drug-taking among youth in Hong Kong.

An early age at first drug use is strongly associated with the risk of developing a substance-use disorder later in life [3]. Youth who persistently misuse substances often have an array of problems, such as academic difficulties, physical and/or mental health-related problems, poor peer relationships, a tendency to exhibit risky sexual behaviour, an increased incidence of blood-borne diseases, and involvement with the juvenile justice system [3–5]. In addition, there are harmful consequences for family members of those who misuse drugs and for their communities and wider society [6]. As a result, youth drug misuse in Hong Kong has become a major problem that cannot be overlooked.

A combination of medical treatment and counselling is the most common treatment for people with drug misuse [7]. However, of the drug-misusing youth surveyed, more than half reported that they had never attempted to abstain from using drugs, only 12.2 % had attempted to seek help, and more than 75 % reported they would not accept medical treatment because they did not believe that they were addicted [2]. Many non-pharmaceutical interventions have previously been applied, but a meta-analysis found that these interventions were ineffective at helping youth stop misusing drugs [7, 8]. A primary reason for this failure was that the patient education methods used may have made drug-misusing youth resistant to accepting interventions [8]. Previous studies have confirmed that recovery from drug misuse is influenced by users’ intentions to change, which are directly determined by their perceptions of anti-drug education and their perceived self-efficacy to change [3]. However, their attitudes, social influences, and demographic characteristics have more indirect effects on recovery from drug misuse [9]. Another reason for the failure of non-pharmaceutical interventions may be that standard interventional content may fail to meet the personal needs of people who misuse drugs. In addition, the meta-analysis also showed that individual interventions comprising more than one session had a stronger effect, whereas brief interventions provided an insufficient number of sessions to support behaviour changes among youth who misuse drugs [8]. Furthermore, qualitative interviews with youth drug misusers have shown that adolescents were reluctant to seek help from sources who they believed would be judgmental, lacked expertise, would reveal their personal information to others, or would inform their parents [10]. These findings demonstrate that feasible, individualised, and effective non-pharmaceutical interventions must be developed to assist Hong Kong’s drug-misusing youth to change their unhealthy behaviour.

### Conceptual framework

Our proposed intervention will be developed with the use and guidance of (1) medical peer-delivered counselling, (2) instant messaging, and (3) brief motivational interviewing.

#### Medical peer-delivered counselling

The use of peer counsellors rather than general counsellors increases the appeal of addiction services to youth and young adult drug users [11]. Because most young people are not eligible to hold positions of power in society, they are subject to authority. This power differential means that communication between adults and youth can be difficult, which manifests as a knowledge gap when attempts are made to engage youth in drug-misuse reduction initiatives. In contrast, the equality in power status between youths means that peer-delivered risk communication is more likely to be successful [11]. In addition, the psychological and social factors associated with the initiation and continuation of drug misuse differ between young people and older adults. Worries about the stigma of drug use, being judged, the exposure of personal information, their parents being informed, not knowing what to expect from the treatment, and major lifestyle changes are the main barriers that prevent youth and young adults from seeking substance use treatment [10, 12]. Youth who misuse substances often experience an array of problems, including academic difficulties, health-related problems (including mental health problems), poor peer relationships, and risky sexual behaviour. Due to their similar age, peers can address the concerns of youths by drawing on their own experiences [13]. Knowledge-sharing and empathetic listening from peers may be more effective at helping drug-misusing youth to overcome or reduce their fear of the unknown regarding the treatment programme [14]. However, addiction counselling on substance use is more complex than counselling for smoking cessation or alcohol and requires more professionalism. Peers with a medical background can therefore best address youths’ concerns, as they have the combined advantages of a similar age and a better ability to master related knowledge and counselling skills than non-medical peers.

#### Instant messaging

Given the ubiquity of mobile technology among youth and the considerable logistical barriers to face-to-face-based care, instant messaging (IM) via mobile applications (apps) such as WhatsApp or WeChat is increasingly being used as a new strategy for health promotion and for enhancement of treatment compliance [13]. A previous study reported that drug-abusing clients preferred to use online counselling because it provided a private and emotionally safe environment that was less confrontational than traditional forms of counselling [15]. Therefore, interaction via smartphone apps encourages those who are reluctant to seek face-to-face help to obtain direct anti-drug misuse advice. IM also enables rapid, real-time interactions, thus delivering continual professional advice and personalised support to help drug misusers overcome drug-withdrawal symptoms.

#### Brief motivational interviewing

Motivational interviewing (MI) was originally developed in the field of alcohol misuse and has been successfully used to treat other health-related behaviours [14, 16]. In contrast to most patient education methods, MI is a direct, client-centred counselling strategy that encourages clients to explore and resolve their ambivalence and promotes their confidence in their ability to change their behaviour. Brief MI has the advantage of saving manpower and, like MI, treats individuals as advocates to initiate and continue their own behavioural change. However, individuals may experience fluctuating motivations before changing their behaviour and thus may do so in a state of ambivalence [17]. Brief MI therefore emphasises the use of shorter and simpler strategies, which include an opening strategy, asking a subject to describe a typical day, exploring the good things and the less good things about exhibiting the targeted behaviour, providing information, comparing the future and the present, exploring concerns, and helping with decision-making. Brief MI uses these strategies as part of a dynamic and interactive process to explore and resolve individuals’ ambivalence and to develop discrepancies between individuals’ core beliefs and the behaviour of not engaging in a desirable health-related lifestyle practice. This process enhances the confidence of individuals and their motivation to change their behaviour.

#### Incorporating medical peer counselling, IM, and brief MI into the promotion of drug misuse reduction among youth

A medical peer-delivered intervention via IM interaction may be more effective in helping to eliminate or reduce drug-abusing youths’ fear of personal-information exposure, in increasing their knowledge of what to expect from treatment and their ability to change their entire lifestyle, and in reducing their worries about withdrawal symptoms. This type of intervention therefore has the potential to increase help-seeking behaviour and the intention to quit among youth who misuse drugs, who are typically reluctant to seek help from services that require real-name registration. Brief MI strategies may be used to guide youth who misuse drugs to attempt a step-by-step process of behaviour change, which may enable them, with assistance, to abstain from drugs.

As stated above, the proposed intervention may be feasible and effective in reducing drug misuse among youth. However, our literature search revealed a paucity of studies that explored the effectiveness of such interventions. This proposed study will therefore involve a randomised controlled trial to determine the effectiveness of a medical peer-delivered intervention that comprises brief MI via IM in reducing drug misuse among youth in Hong Kong.

## Methods

### Study design

**Fig. 1:**
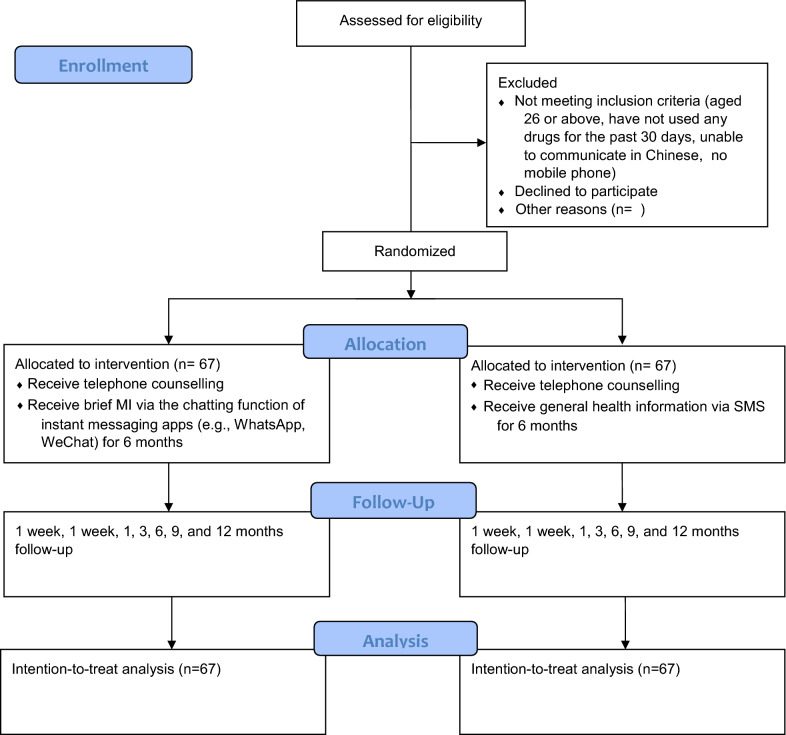
CONSROT Flow diagram

This proposed study will be a two-arm, parallel-group, pragmatic randomised controlled trial (RCT) with a 1:1 allocation ratio. It will compare the reduction in drug misuse at 12 months in two groups of youths: those who are individually randomised to participate in the intervention group, who will receive medical peer-delivered, interactive brief MI via IM; and those in the control group, who will receive general health information via text messaging (Fig. 1).

### Resource and setting

This proposed study will be conducted in association with the Medical Peer Addiction Counselling Quitline Service (MedPAC Quitline), supported by the Beat Drugs Fund, Narcotics Division, Security Bureau of Hong Kong. The team of the School of Nursing at the University of Hong Kong established this first peer-oriented drug misuse quitline in 2020. Trained youth and young adult counsellors with a medical background provide peer-oriented drug-misuse counselling via MedPAC Quitline to drug-abusing youth and young adults and actively refer those in need to further treatment and rehabilitation services.

### Participants and sampling

Callers to the MedPAC Quitline and those who are actively recruited from schools and communities will be eligible service targets for the MedPAC Quitline. Youth who misuse drugs and have contacted the MedPAC Quitline will be eligible for inclusion in this proposed study if they (1) are 25 years of age or younger, (2) report having taken drugs within the past 30 days, (3) are able to communicate in Chinese, (4) agree to receive counselling via a messaging app (such as WhatsApp or WeChat), and (5) verbally consent to join the follow-up intervention. We will exclude potential subjects who have communication barriers and those who are undergoing other drug-misuse recovery treatments.

Because no previous study has conducted a brief telephone-based MI intervention, the sample size for the proposed RCT has been calculated according to a previous RCT that involved a computer-based MI intervention [18]. In this previous RCT, the mean number of drug-misuse consumptions per day decreased from 11.94 at baseline to 5.52 at 12 months for participants in the intervention group, and 9.22 at baseline to 8.61 at 12 months for those in the control group. The effect size was 0.64 for the MI intervention on drug-misuse reduction, with reference to the control group [18]. Based on these results, to detect a two-sided significant difference between groups with a power of 80 % and a significance level of 5 %, which are commonly accepted metrics for behavioural studies, the required sample size for this proposed RCT has been estimated to be 40 participants in each group. Given an expected retention rate of approximately 60 % at the 12-month follow-up, the target number of participants will be at least 134 (67 per group).

### Recruitment

Recruitment will begin in March 2021. The MedPAC Quitline Service is promoted to Hong Kong residents through a variety of platforms, such as publicity and exhibitions in secondary schools and tertiary educational institutions, non-governmental organisations, and industries; community outreach activities; advertisements in newspapers, magazines, and on the Internet; the MedPAC Quitline website and social media platforms; posters; and snowball approaches. At least 20,000 pamphlets, booklets, and other publicity materials as well as invitation letters for this study will be delivered to secondary schools and tertiary educational institutions, including middle schools, vocational training schools, colleges, and universities. Exhibitions, carnivals, and anti-drug games and competitions will be held in secondary schools and tertiary educational institutions that agree to participate in this project. Our research assistant will screen all potential subjects and will invite those who meet the inclusion criteria to participate in the study.

### Randomisation and blinding

A simple complete randomisation method will be adopted. The subjects will be randomly allocated to either the intervention or the control group. The random numbers used for group assignment will be generated using a personal computer by another research assistant, who will not be involved in the subject recruitment. The randomisation will be performed by a research assistant who will open a serially numbered, opaque, sealed envelope that contains a card indicating the randomly allocated group. Because the subjects will be aware of their allocation according to the content received, we will be unable to completely blind them. However, a single-blind approach will be adopted, in which all outcome assessors and data analysts will be blinded to the group assignment.

### Intervention

In addition to receiving the basic service provided by the MedPAC Quitline, the subjects in the intervention and control groups will receive brief MI via IM interaction and general health text-messages, respectively.

#### Intervention group

The interaction communication using brief MI will be applied via the chat function of IM apps (e.g., WhatsApp or WeChat). The intervention will be performed for 6 months at a frequency of at least twice a week. To begin the conversation, trained peer counsellors will actively contact the participants and explore their lifestyles and their current barriers to and facilitators of change. The counsellors will do this by asking participants about their current lifestyles, drug-misuse habits, and levels of stress to establish a collaborative relationship with and obtain a general understanding of each participant (opening strategy). To further understand why a participant does not intend to reduce his/her drug misuse, the counsellor (interventionist) will review a typical day of the subject (a typical day) and ask about the advantages and disadvantages of not reducing drug misuse. Each participant will receive further information about the health benefits of reducing or quitting drug use, if he/she is interested in doing so (providing information). To elicit information on discrepancies, each participant will be encouraged to talk about his/her ideal future and to compare the past and the present (the future and the present). The interventionist will also listen to, explore, and summarise the concerns of the participant (exploring concerns). The interventionist will resolve the ambivalence of the participant by describing what others have done in a similar situation and presenting options, while not urging a participant to make a decision (helping with decision*-making*). Importantly, the participant’s confidence about reducing or quitting his/her drug use will be enhanced throughout the process of receiving personalised health advice, as this advice will emphasise the health benefits of reducing or quitting and encourage the participant to be aware of resources that enable and facilitate such behavioural change. At the request of the participant, the interventionist will provide more comprehensive information and suggestions on how to reduce drug use, including the skills to overcome withdrawal symptoms.

#### Control group

The subjects will receive general health information via text message, such as ‘do physical exercise for at least 30 minutes per week to keep yourself healthy’, for 6 months at a frequency of twice a week.

### Measures

The demographics and baseline characteristics of participants will be collected during the baseline phone counselling. A structured questionnaire will be developed for this purpose by adopting or modifying international and/or locally validated instruments. The questionnaire will gather information on a participant’s (a) drug misuse profile, including the types of drugs used, his/her number of days of use, his/her daily drug consumption, and his/her history of quitting; (b) beliefs and values relating to drug use, using the Chinese Drug Involvement Scale [19]; (c) drug misuse-related knowledge [20]; (d) attitudes towards their drug abuse [20]; (e) contemplation stage of drug-misuse abstinence, using the Contemplation Ladder Tool [21]; (f) treatment needs and motivation, using the Texas Christian University Motivation and Readiness For Treatment form [22]; (g) self-efficacy to avoid drug use, using the Adolescent Relapse Coping Questionnaire [23]; (h) quality of life, using the EuroQol 5-Dimension 5-Level Questionnaire [24]; (i) depression level, using the Center for Epidemiologic Studies Depression Scale [25]; and (j) self-esteem, using the Rosenberg Self-Esteem Scale [26]. The Chinese versions of the above scales have been evaluated to show acceptable validity and reliability [19–26].

All participants will receive follow-up telephone calls from our trained research assistant at 1 week, 1 month, and 3, 6, 9, and 12 months. During each follow-up telephone call, the participants will be asked to complete the structured questionnaire (Table 1). Participants who are lost to one follow-up will be contacted again in the next follow-up.

**Table 1:** Schedule of enrollment and follow-up assessments

Assessment	Time-point						
	Baseline	1 week	1 Month	3 Months	6 Months	9 months	12 Months
Informed consent for participants	×						
Eligibility screen for participants	×						
Telephone counselling	×						
Sociodemographic characteristics	×						
Randomization	×						
Intervention	× (twice per week)						
Self-reported drug misuse	×	×	×	×	×	×	×
Attempt to quit	×	×	×	×	×	×	×
Chinese drug involvement scale	×				×		×
Drug misuse-related knowledge	×				×		×
Attitudes towards the drug misuse	×				×		×
Contemplation ladder	×	×	×	×	×	×	×
TCU MOTForm	×			×	×	×	×
Adolescent relapse coping questionnaire	×			×	×	×	×
EQ-5D-5 L level	×				×		×
Center for Epidemiologic Studies Depression Scale	×				×		×
Rosenberg self-esteem scale	×				×		×
Referral need and results	×	×	×	×	×	×	×

### Outcomes

The primary outcome will be changes in the participants’ reductions in self-reported drug consumption at 12 months compared to that at baseline. Secondary outcomes will be (1) changes in the participants’ reductions in self-reported drug consumption at 6 months compared to that at baseline; (2) 30 days self-reported drug abstinence at 6 and 12 months; (3) changes in relapse risk among the quitters at 6 and 12 months compared to that at baseline; (4) changes in the participants’ contemplation stages at 6 and 12 months compared to those at baseline; and (5) treatment needs and motivation at 6 and 12 months compared to those at baseline.

### Data analysis

Data analysis will be performed using the Statistics Package for the Social Sciences (SPSS) version 25.0 for Windows (SPSS Inc., Chicago, IL, USA). Analyses will be conducted on an intention-to-treat basis, with missing-value substitutions based on the last observation carried forward. A two-sided statistical significance level of 0.05 will be adopted for all analyses.

Descriptive statistics will be used to detail participants’ demographic characteristics and drug misuse profiles. Frequencies and percentages or means and standard deviations will be used to present categorical data and continuous data, respectively. The differences between intervention and control groups, in terms of sociodemographic factors and outcomes, will be compared using a chi-square test for categorical variables and by an analysis of variance for continuous variables.

#### Main analysis

Odds ratios, risk differences, and 95 % confidence intervals will be used to compare the primary and secondary outcomes between the two trial groups. A generalised estimating equation model-based analysis will be used to summarise the intervention effect on the primary and secondary outcomes with and without adjustment characteristics, referral to a treatment institution, and the interaction times.

#### Exploratory analysis

A subgroup analysis of the participants in the intervention group will be conducted using different IM apps and data on referrals to treatment institutions to determine whether the relative effects of study treatments vary significantly among subgroups of IM app users. Propensity score matching, including demographics and types of abused drugs, will be used to further determine the true cause-and-effect relationship between the intervention and control groups, with restrictions of these subgroups. Missing outcomes will be assumed to be dependent on observed data (missing at random), and the observed data will be analysed with a multiple imputation procedure to impute the missing values, as a sensitivity analysis.

#### Text mining of IM app group conversations

All interaction content will be archived and anonymised to remove identifying personal information. Due to the large number of messages that will be collected from the IM apps, we will use automatic, computational text-mining and visualisation of the dataset for the content analysis. First, using a lexicon of keywords derived from our qualitative analysis of the pilot RCT (unpublished), we will develop a heat-map visualisation to illustrate the prevalence of the discussion topics. Second, we will apply topic modelling to investigate emerging themes in our content dataset, using the Mallet [27] implementation of the Latent Dirichlet Allocation topic modelling algorithm [28]. Topic modelling algorithms use a text dataset (in this case, the content dataset) as input and then output a set of topics (and their associated keywords) in addition to estimates of the proportion of each topic [29].

## Discussion

Four caveats should be considered when the findings of this proposed study are interpreted. First, this pragmatic RCT will not guarantee ‘full’ intervention compliance by all participants. For instance, the participants in the intervention group will be encouraged to interact with the interventionists, but they will have the right to ignore the IMs. Thus, they will be able to choose to either respond to an interventionist or to remain silent throughout the intervention period. As a result, although the RCT will show the effectiveness of delivering such an interaction for drug-misuse cessation, we expect that there will be a variety of levels of participation in the interaction. Nevertheless, all IM conversations will be archived for further content analysis and compliance analysis. The actual effect of participating in an interaction will be investigated by filtering out participants who exhibit low participation. Second, we will only recruit drug-misusing youths in this RCT who have also enrolled in the MedPAC Quitline service, and thus the findings will not be generalisable to unassisted youths who misuse drugs. Future trials that customise the intervention for drug-misusing youths recruited from outside the service will be warranted. Third, recreational users, who are common among young people with substance use, will be also included in this trial. This could potentially limit the room for significant improvement. Considering recreational drug use is a common situation among the youth population, this group should not be ignored. To explore the effect of the intervention in the different participants with such use patterns, we will conduct post hoc analysis for subgroups of the pattern of use, dependence level, readiness to quit in the next 30 days, and previous quit history at baseline. Fourth, all measures will be self-reported. However, the validity of self-reported versus biochemical assessment of substance use may vary according to a variety of contextual factors. Evidence of the ability of existing biochemical methods to detect reductions in drug misuse remains scarce. Nevertheless, a previous study indicated that self‐report may outperform biological assessment for the assessment of patterns of drug use over the past month, because biological assessment typically only captures substance use that has occurred in the past 5 days [30].

The findings from this study will be disseminated locally and internationally through manuscript publications in peer-reviewed journals and conference presentations at national and international platforms. The proposed intervention has the potential to increase the help-seeking behaviour and intention to quit of youth who misuse drugs and are reluctant to seek help from services that require real-name registration. With such assistance, more youth may abstain from drug misuse. This proposed study will be the first RCT to assess the effectiveness of a medical peer-delivered, IM-based, interactive intervention in reducing drug misuse among youth in Hong Kong. Thus, this study will inform decisions on whether it is worthwhile to invest resources in large-scale implementation of such an intervention to reduce drug misuse in Hong Kong’s youth.

## Data Availability

The data that support the findings of this study will be available f from the authors upon reasonable request and with permission of the corresponding author William HC Li (William3@hku.hk ).

## Abbreviations

IM: Instant Messaging
MI: Motivational interviewing
RCT: Randomised Controlled Trial
MedPAC: Medical Peer Addiction Counselling
SPSS: Statistics Package for the Social Sciences
